# Modeling Floral Induction in the Narrow-Leafed Lupin *Lupinus angustifolius* Under Different Environmental Conditions

**DOI:** 10.3390/plants13243548

**Published:** 2024-12-19

**Authors:** Maria A. Duk, Vitaly V. Gursky, Mikhail P. Bankin, Elena A. Semenova, Maria V. Gurkina, Elena V. Golubkova, Daisuke Hirata, Maria G. Samsonova, Svetlana Yu. Surkova

**Affiliations:** 1 Mathematical Biology and Bioinformatics Laboratory, Peter the Great Saint Petersburg Polytechnic University, 195251 St. Petersburg, Russia; 2 Theoretical Department, Ioffe Institute, 194021 St. Petersburg, Russia; 3 Faculty of Agronomy and Ecology, Far Eastern State Agrarian University, 675005 Blagoveschensk, Russia; 4 Astrakhan Experiment Station, N.I. Vavilov All-Russian Institute of Plant Genetic Resources, 416462 Astrakhan, Russia; 5 Department of Genetics and Biotechnology, Saint-Petersburg State University, 199034 St. Petersburg, Russia

**Keywords:** *FT*-like genes, meristem identity genes, gene expression, floral induction, legumes, narrow-leafed lupin, *Lupinus angustifolius*, vernalization, photoperiod, mathematical modeling

## Abstract

Flowering is initiated in response to environmental cues, with the photoperiod and ambient temperature being the main ones. The regulatory pathways underlying floral transition are well studied in *Arabidopsis thaliana* but remain largely unknown in legumes. Here, we first applied an in silico approach to infer the regulatory inputs of four *FT*-like genes of the narrow-leafed lupin *Lupinus angustifolius*. We studied the roles of *FTc1*, *FTc2*, *FTa1*, and *FTa2* in the activation of meristem identity gene *AGL8* in response to 8 h and 16 h photoperiods, vernalization, and the circadian rhythm. We developed a set of regression models of *AGL8* regulation by the *FT*-like genes and fitted these models to the recently published gene expression data. The importance of the input from each *FT*-like gene or their combinations was estimated by comparing the performance of models with one or few *FT*-like genes turned off, thereby simulating loss-of-function mutations that were yet unavailable in *L. angustifolius*. Our results suggested that in the early flowering *Ku* line and intermediate *Pal* line, the *FTc1* gene played a major role in floral transition; however, it acted through different mechanisms under short and long days. Turning off the regulatory input of *FTc1* resulted in substantial changes in *AGL8* expression associated with vernalization sensitivity and the circadian rhythm. In the wild *ku* line, we found that both *FTc1* and *FTa1* genes had an essential role under long days, which was associated with the vernalization response. These results could be applied both for setting up new experiments and for data analysis using the proposed modeling approach.

## 1. Introduction

Flowering is controlled by a large number of signaling pathways providing the developmental regulation and response to environmental conditions. Major factors affecting the timing of floral transition include the photoperiod and vernalization [1,2].

**Figure 1:**
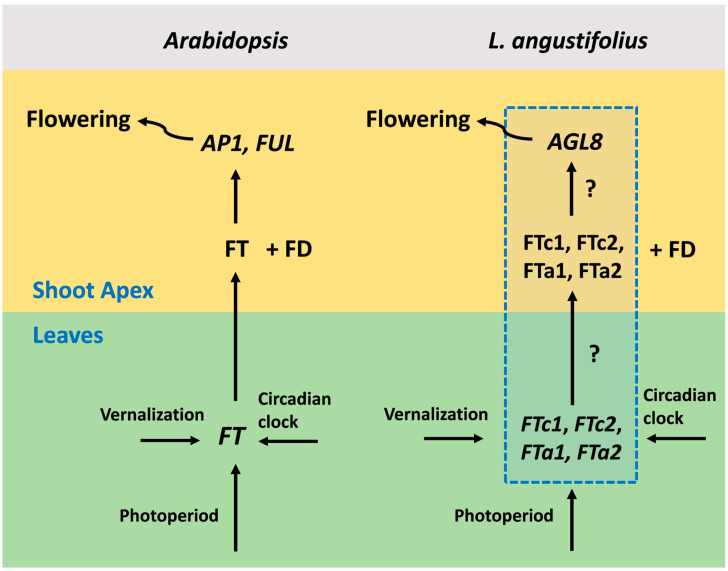
A general scheme of flowering initiation in *Arabidopsis thaliana* and a putative network in the narrow-leafed lupin *Lupinus angustifolius*. In *Arabidopsis*, the expression of the *FT* gene is activated in the leaves by the photoperiod and vernalization pathways. Next, the FT protein becomes expressed in the shoot apical meristem, where in complex with the transcription factor FD, it activates meristem identity genes, including *AP1* and *FUL*. Meristem identity genes, in turn, activate pathways responsible for the formation of floral organs. *L. angustifolius* has four *FT* gene orthologues, which are *FTc1*, *FTc2*, *FTa1*, and *FTa2*. The mechanisms of *FT*-like gene activation by environmental signals and the involvement of each *FT*-like gene in the regulation of meristem identity genes are still unknown (shown in the blue dotted box). *AGL8* is the *L. angustifolius* orthologue of the *Arabidopsis AP1* and *FUL* genes and a putative target of *FT*-like genes.

The detailed mechanisms of flowering regulation have been revealed in *Arabidopsis thaliana* [3,4]. The main floral activator and integrator of various signaling pathways is the *FLOWERING LOCUS T* (*FT*) gene, whose expression is turned on in response to environmental signals in leaves. The small mobile FT protein moves through the phloem to the shoot apex, where in complex with the transcription factor FD, it activates meristem identity genes such as *APETALA1* (*AP1*), *LEAFY* (*LFY*), and *FRUITFULL* (*FUL*) [5,6,7] (Figure 1). Meristem identity genes control the formation of floral organs [8]. The major regulators of photoperiod and vernalization in *Arabidopsis* are *CONSTANS* (*CO*) [9,10] and *FLOWERING LOCUS C* (*FLC*) [11], whose protein products bind directly to the promoter region and first intron of the *FT* gene, respectively [12,13,14]. In the non-inductive conditions, FLC represses *FT* in a complex with SHORT VEGETATIVE PHASE (SVP) [15]. The vernalization treatment switches the mechanism of *FLC* silencing, leading to *FT* de-repression, which becomes activated by the CO protein. CO is also responsible for the circadian clock control, resulting in the rhythmic expression of *FT* during the day time [12,16,17]. *FT*-like genes promote flowering in most plant species [18,19].

In legumes, the mechanisms of flowering induction in response to environmental signals appear to be more complex than in *Arabidopsis* for several reasons. First, orthologs of the *FLC* and *CO* integrator genes are absent or inactive in vernalization-sensitive legume species [20,21]. Secondly, the genomes of temperate legumes contain four to six *FT*-like genes, grouped into three subclades, namely *FTa*, *FTb*, and *FTc* [22]. Although the upstream regulators of the *FT*-like genes have not yet been identified in many legume species, multiple studies have suggested that these genes are the major targets of vernalization and photoperiod signals [23,24,25]. However, the mechanisms by which several *FT*-like genes act as floral integrators also remain largely unexplored. It has been shown that the *FT*-like genes in legumes differ in their function, with one or two genes being most critical in their regulatory role. For example, in *Medicago trancatula*, these genes are *FTa1* and *FTb1*, and in *Pisum sativum*, *FTb2* and *FTa1* [22,23].

The narrow-leafed lupin *Lupinus angustifolius* is a valuable legume crop with high grain protein content and substantial contribution to soil improvement. It has four *FT*-like genes, which are *FTa1*, *FTa2*, *FTc1*, and *FTc2* [24]. The major gene responsible for the vernalization-induced flowering in *L. angustifolius* is *FTc1* [24,26]. The natural mutations that occurred in this locus during the domestication period resulted in early flowering and loss of vernalization insensitivity. Two deletions in cultivated varieties encompassing 1423 bp and 5162 bp of the *LanFTc1* promoter region were named as *Ku* and *Jul*, respectively, while the wild allele having intermediate phenology and carrying 1208 bp deletion was named as *Pal*. The wild allele (*ku*) without a mutation retained vernalization responsiveness and flowered late [21,26].

The recently published *L. angustifolius* dataset [21] includes the expression of four *FT*-like genes and their putative target *AGL8*, whose protein sequence revealed the highest similarity to *A. thaliana FUL*/*AGL8* (AT5G60910) and *AP1* (AT1G69120) genes [21]. The expression data varied with respect to vernalization, the photoperiod, and circadian clock. The data were obtained for three alleles (*Ku*, *Pal*, and *ku*) having different flowering times and vernalization sensitivity. This set of genes can provide a complete description of the flowering initiation, since each of them acts as an integrator of a large number of signaling pathways. Thus, the expression of each integrator reflects the influence of many genes (Figure 1). Such a “hub” approach has been earlier applied in multiple modeling studies that inferred regulatory interactions underlying floral transition [27]. Thus, we used this dataset to model the influence of vernalization, the photoperiod, and circadian rhythm on flowering initiation in the narrow-leafed lupin.

The mathematical modeling of gene networks is a powerful tool to predict regulatory mechanisms based on the dynamics of gene expression. Earlier studies on modeling floral transition in plants were mainly conducted in *Arabidopsis* [28,29,30,31]. A number of publications have presented in silico analyses of flowering networks in pea (*P. sativum*) [32], chickpea (*Cicer arietinum*) [33], and *M. trancatula* [34]. However, none of the previously published models of floral transition networks in legumes considered the effect of vernalization under different photoperiod lengths and times of day.

The following questions remain unanswered: (1) if there is a “major” vernalization response gene, do other *FT*-like genes play any role in vernalization-induced flowering? (2) How does each *FT*-like gene regulate meristem identity genes? (3) How does the regulation by *FT*-like genes vary with respect to the photoperiod, vernalization, and circadian clock? (4) How do the regulatory mechanisms change in the accessions with different flowering times and vernalization sensitivities? Each question is common to all legumes and none of these issues have yet been studied in detail.

In this paper, we constructed a set of linear regression models to predict the regulatory mechanisms of flowering initiation in *L. angustifolius*. First, we selected the best model structure of *AGL8* regulation by *FT*-like genes. Next, we applied this model to experimental data and considered expression patterns of *AGL8* resulting from its activation by different combinations of *FT*-like genes. We excluded the regulatory inputs of *FT*-like genes one by one and evaluated the *AGL8* patterns in the models based on (1) the cost function values, (2) specific discrepancies between gene expression patterns in the model and experiment, and (3) values of the regulatory parameters.

Our models showed that in the early flowering *L. angustifolius Ku* line and in the *Pal* line with intermediate phenology, turning off the regulatory input of *FTc1* resulted in changes in *AGL8* expression associated with vernalization sensitivity and circadian rhythm, which differed between short and long days. In the wild *ku* line, *FTa1* and *FTc1* genes played the most prominent role during long days, which was dependent on vernalization. Our models did not predict any specific function of *FTa2* and *FTc2* genes in *AGL8* regulation in all *L. angustifolius* lines.

Overall, here we first used mathematical modeling to predict regulatory interactions underlying an influence of vernalization and the photoperiod on floral induction in the narrow-leafed lupin. The suggested approach can be applied to other legume species once the experimental data become available.

## 2. Results

### 2.1. Selection of Model Structure for AGL8 Regulation

Our aim was to test how *FT*-like genes might contribute to *AGL8* activation in the narrow-leafed lupin using gene expression data models. The data included the relative mRNA concentrations of five *L. angustifolius* genes (*FTa1*, *FTa2*, *FTc1*, *FTc2*, and *AGL8*) in the following three lines: the line carrying the domesticated early flowering *FTc1* allele (*Ku*), the line carrying the wild allele *ku*, and the line carrying the *Pal* allele, which is an intermediate between the domesticated and wild lines [21]. The data were obtained for 8 h (SD) and 16 h (LD) photoperiods with and without vernalization at two time points during the day, at 9 A.M. and 3 P.M. for SD and 7 A.M. and 6 P.M. for LD (see “Materials and Methods”) [21]).

An important problem in modeling is finding a balance between the complexity of the model structure, expressed, for example, in the number of free parameters, and the level of data diversity, expressed in data type and amount. Too complex models are prone to overfitting, while too simple ones may not be useful for testing meaningful hypotheses about underlying mechanisms. We utilized linear regression as a modeling framework that is simple and suitable for the available data and discarded modeling based on ordinary differential equations due to poor temporal resolution. Therefore, the *AGL8* expression level was represented as a linear combination of the *FT*-like gene expression levels at each time point. *AGL8* expression in the model was fitted to that in the data by minimizing the cost function F (see “Materials and Methods”).

The simplest model of *AGL8* regulation (**Model 1**) was formulated under the assumption that *FT*-like genes have the same regulatory parameters:

$$AGL8=c_{0}+c_{1}\sum_{i}{FT}_{i},$$

where constants *c_i_* can be interpreted as regulatory parameters, with *c*_0_ including the contribution of non-FT factors to the regulation of *AGL8* and *c*_1_ reflecting the cumulative regulatory inputs of all *FT*-like genes.

The basal expression level quantified by the coefficient *c*_0_ should adequately represent different conditions in the data, so it was assumed to take different values for different conditions (vernalization conditions and circadian rhythm). Since the focus of our study was on the *FT*-like genes in *L. angustifolius* lines with different vernalization sensitivities, we also allowed *c*_1_ to vary between vernalized and non-vernalized conditions. We examined that this assumption on *c*_1_ was informative by comparing the model performance on the SD expression data with and without this assumption. As an additional test, we calculated the model performance on data under the assumption that *c*_1_ varied between the morning and the evening data. Results showed that variation in *c*_1_ across vernalization conditions substantially improved the model performance for the intermediate *Pal* line, which partially retains vernalization sensitivity, and it was not so effective for the vernalization-insensitive *Ku* line (Supplementary Figure S1). Moreover, variation in *c*_1_ across times of the day did not produce an essential difference in the model performance on the expression data of both lines. Therefore, implementing different vernalization conditions via the variation in parameter *c*_1_ for these conditions was informative and thus was kept in all models considered in the study.

As alternatives to **Model 1**, we also tested two models with separate regulatory parameters for different *FT*-like genes.

In **Model 2**, the ${FT}_{c1}$ gene was singled out as the “major” regulator, while ${FT}_{a1}$, ${FT}_{a2}$, and ${FT}_{c2}$ had the same regulatory parameters:

$$AGL8=c_{0}+c_{1}{FT}_{c1}+c_{2}\sum_{i\neq c1}{FT}_{i}.$$

In **Model 3**, all *FT* genes had their own regulatory parameters:

$$AGL8=c_{0}+c_{1}{FT}_{c1}+{c_{2}FT}_{a1}+c_{3}{FT}_{c2}+{c_{4}FT}_{a2}.$$

**Models 2 and 3** are convenient for testing hypotheses about different regulatory roles of *FT*-like genes in *AGL8* regulation, but whether we can use them depends on whether these models outperform **Model 1** on data considering different numbers of free parameters in the models.

To select the best model structure, we fitted Models 1–3 to experimental data from the 8 h dataset with and without vernalization collected at different times of the day. At this step, we considered only SD data, since the LD data had only 2–3 points in dynamics, which was not enough for rigorous model comparison.

**Figure 2:**
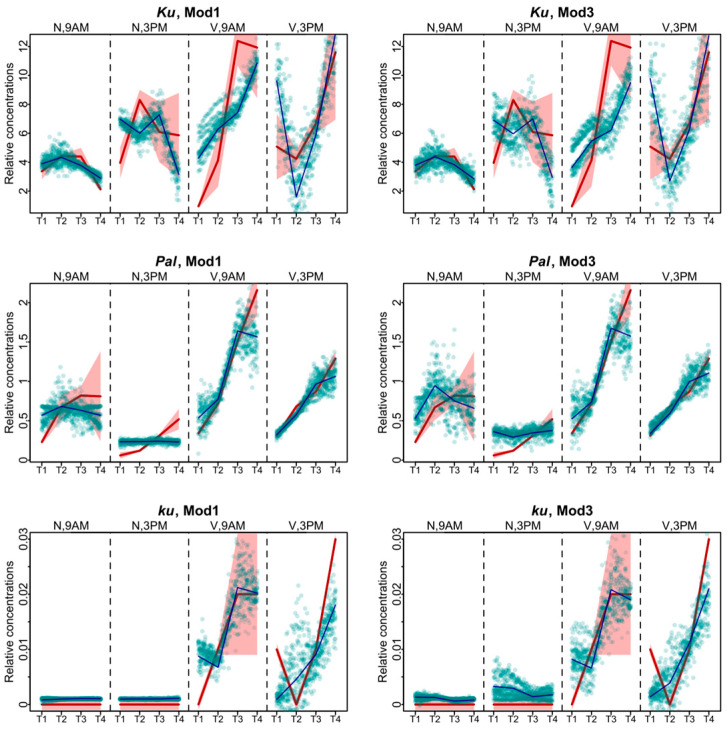
Data fitting results for Models 1 and 3, which show the lowest values of the cost function. Averaged dynamics and standard deviation of the experimental data are shown in red, and the model solutions (averaged over 1000 runs) are shown in black. Green dots represent the simulation results from 10 randomly chosen runs of the minimization process. “N” and “V” stand for non-vernalized and vernalized data, respectively. “9 A.M.” and “3 P.M.” are the times of the day when the data were collected. T1–T4 stand for sampling terms [21].

**Figure 3:**
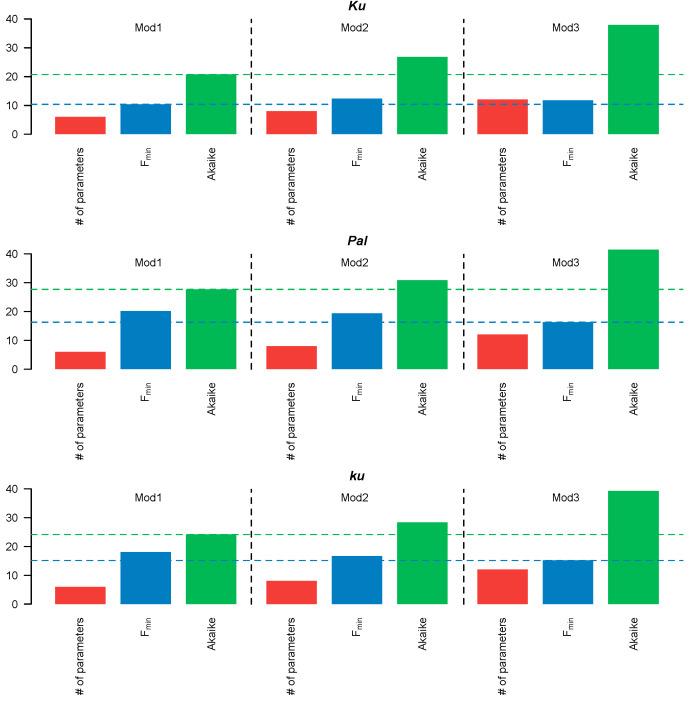
Number (#) of free parameters (red), minimal cost function value F_min_ (blue), and AIC value (green) for Models 1–3. The blue and green dotted lines correspond to the minimum values of the cost function and AIC, respectively.

The models produced largely similar fits (Figure 2), with the best values of the cost function being in Models 1 and 3 (Figure 3). For the *Ku* line, the solutions of these two models differed mostly for the vernalized data. The solution of **Model 1** was closer to experimental data at 9 A.M., while **Model 3** better reproduced the data at 3 P.M. For *Pal*, the fits differed mostly in the non-vernalized data, with **Model 1** exhibiting the more relevant dynamics. For the *ku* line, differences between the two models were observed at 3 P.M. At that time, **Model 1** performed better for the data without vernalization, while **Model 3** for the vernalized data (Figure 2).

Since **Models 1–3** had different numbers of free parameters, we compared their performance using the Akaike information criterion (AIC), which accounts for both the fitting quality and the number of free parameters. The simplest, **Model 1**, in which all *FT*-like genes acted cumulatively, exhibited the lowest AIC values across all *L. angustifolius* lines (Figure 3). Therefore, the more complicated structures of Models 2–3 were not justified, so we selected **Model 1** for further analysis.

### 2.2. Modeling Regulatory Inputs of FT-like Genes to AGL8 Activation

To predict the importance of each *FT*-like gene in *AGL8* activation, we conducted a number of numerical experiments. We designed a set of models based on the selected **Model 1** by excluding one or several *FT*-like genes from the total sum over *FT*-like genes in the model equation. Depending on which *FT*-like genes were excluded, these models aimed to test the following hypotheses:

**Hypothesis** **H0.** *all FT-like genes are involved in AGL8 regulation (pure **Model 1**).*

**Hypothesis** **H1.** *FT_a2_ is not involved in AGL8 regulation:*

$$AGL8=c_{0}+c_{1}\left({FT}_{c1}+{FT}_{c2}+{FT}_{a1}\right)$$

**Hypothesis** **H2.** *FT_a1_ is not involved in AGL8 regulation:*

$$AGL8=c_{0}+c_{1}\left({FT}_{c1}+{FT}_{c2}+{FT}_{a2}\right)$$

**Hypothesis** **H3.** *FT_c2_ is not involved in AGL8 regulation:*

$$AGL8=c_{0}+c_{1}\left({FT}_{c1}+{FT}_{a1}+{FT}_{a2}\right)$$

**Hypothesis** **H4.** *FT_c1_ is not involved in AGL8 regulation:*

$$AGL8=c_{0}+c_{1}\left({FT}_{c2}+{FT}_{a1}+{FT}_{a2}\right)$$

**Hypothesis** **H5.** *AGL8 expression can be explained solely by the regulation of the FT_c1_ gene:*

$$AGL8=c_{0}+c_{1}{FT}_{c1}.$$

**Figure 4:**
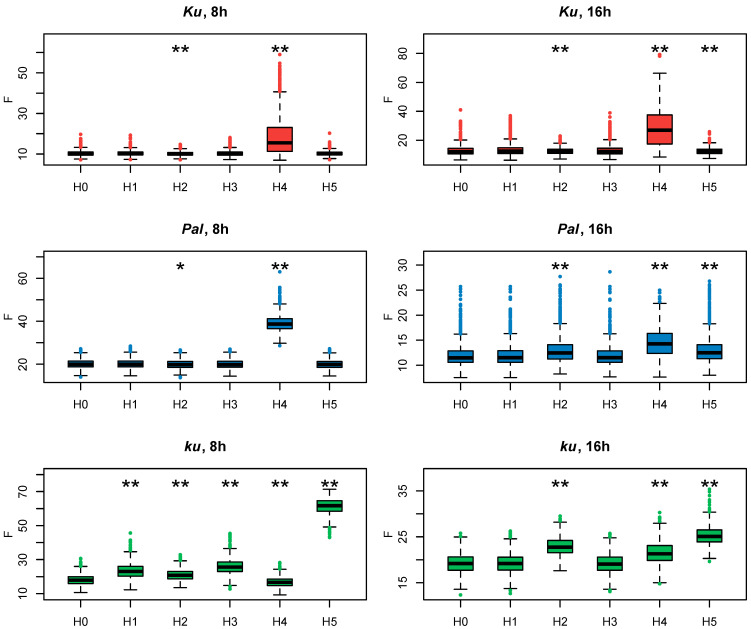
Cost function values (F) from 1000 minimization runs in **Model 1** under hypotheses H0–5 for three *L. angustifolius* lines. Asterisks indicate statistically significant differences in the mean F between Hi (i = 1…5) and H0 (* *p* < 0.05, ** *p* < 0.01). The labels 8 h and 16 h are SD and LD photoperiods, respectively.

Since all these models had the same number of parameters, we evaluated how they fit to experimental data according to the cost function values (Figure 4). If the exclusion of an *FT*-like gene from the model led to a substantial decrease in the model performance on the *AGL8* expression data, that would suggest the importance of this gene in *AGL8* regulation.

#### 2.2.1. Analysis of *AGL8* Regulation for 8 h Photoperiod

To understand which *FT*-like gene mostly affects *AGL8* regulation, we compared the cost function values of Models H1–H5 with that of **Model 1** under the null hypothesis H0 (**Model H0**).

In *Ku* and *Pal* lines, the F values of Models H1, H3, H5, and H0 did not differ significantly (Figure 4). A small but statistically significant difference was detected between the F values of **Model H2**, where *FTa1* was excluded from *AGL8* regulation, and **Model H0**. A substantially increased value of the cost function was produced only in **Model H4**, without the input of *FTc1*, which suggested the importance of this gene in this case. Interestingly, regulation by *FTc1* alone (**Model H5**) did not affect *AGL8* expression in *Ku* and *Pal*, showing that this gene could act as a sole activator of *AGL8* (Figure 4) On the contrary, in the late flowering vernalization-sensitive *ku* line, **Model H5** produced the worst fits, while **Model H4** had the same fit quality as H0. In the *ku* line, we detected the statistical difference between the values of the cost function of Models H1–4 and **Model H0**, but their mean values were not essentially different from that in **Model H0** (Figure 4).

These results demonstrated that under the short days in the *Ku* and *Pal* lines, *AGL8* activation mostly depended on *FTc1*, while in the wild *ku* line, *FTc1* was unable to fully provide *AGL8* activation without an input from other *FT* genes.

#### 2.2.2. Analysis of *AGL8* Regulation for 16 h Photoperiod

As in the models for short days, in the case of the 16 h photoperiod data for the *Ku* and *Pal* lines, the highest value of the cost function was found in **Model H4** (Figure 4). In the *Pal* line, there was a statistically significant difference in the cost function value between **Model H0** and **Models H2 and H5**, suggesting the involvement of more *FT*-like genes in the *AGL8* regulation under LD. In the *Ku* line, the cost function values of Models H0, H2, and H5 were very similar, despite the small p-value (Figure 4). Thus, in the *Ku* and *Pal* lines, the exclusion of *FTc1* from *AGL8* regulation mostly affected the F values under both 8 h and 16 h photoperiods.

In contrast, for the wild *ku* line, our models suggested different regulations between short and long days. In both photoperiods, the fits in **Model H5** were much worse than in **Model H0**. However, in contrast to SD, the cost function values of Models H2 and H5 also showed a statistically significant difference and had substantially higher values than in **Model H0** (Figure 4). Thus, our models suggested that the *FTa1* and *FTc1* genes played an essential role in *AGL8* regulation in the *ku* line under the 16 h photoperiod.

#### 2.2.3. *AGL8* Pattern Defects in Models Vary with Respect to Vernalization and Circadian Rhythm

**Figure 5:**
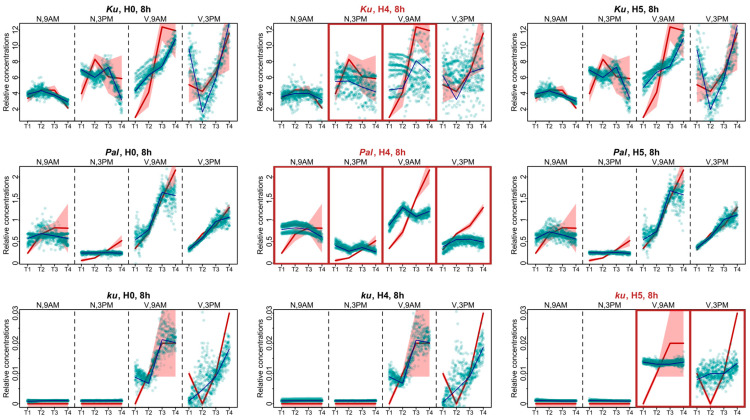
Expression dynamics in **Models H4, H5, and H0** compared to experimental data for the 8 h photoperiod. Averaged dynamics of the experimental data are shown in red, and the model solution (average of 1000 runs) is shown in blue. Green dots represent the simulation results from 10 random runs of the minimization process. Models showing specific defects in solutions compared to H0 are marked with brown frames. “N” and “V” stand for non-vernalized and vernalized data, respectively. The labels “9 A.M.” and “3 P.M.” are the times of the day when the data were collected. T1–T4 stand for sampling terms [21].

Another way to investigate the influence of *FT*-like genes within the framework of **Model 1** is to analyze specific defects in model solutions and compare these defects across the models with all *FT*-like genes present and with some *FT*-like genes excluded. Therefore, we examined the *AGL8* expression patterns in Models H4 and H5, which had the worst cost function values, and compared them to **Model H0**. During the 8 h photoperiod, for all *L. angustifolius* lines, **Model H0** reflected the overall dynamics of *AGL8* expression, although with some uncertainties mainly affecting the first and last time points (Figure 5).

The lack of regulatory influence of the *FTc1* gene in **Model H4** led to the impairment of all fits in the *Pal* line. The *AGL8* expression patterns after vernalization were mostly affected, where the model did not reproduce an increase in expression levels prior to flowering (Figure 5). For the wild *ku* line, we also detected similar defects in the fits of **Model H5** to the vernalized data (Figure 5). The patterning defects in the *Ku* line were less pronounced compared to **Model H0**. The worst fits of **Model H4** were detected for the non-vernalized data at 3 P.M. and the vernalized data at 9 A.M. (Figure 5).

**Figure 6:**
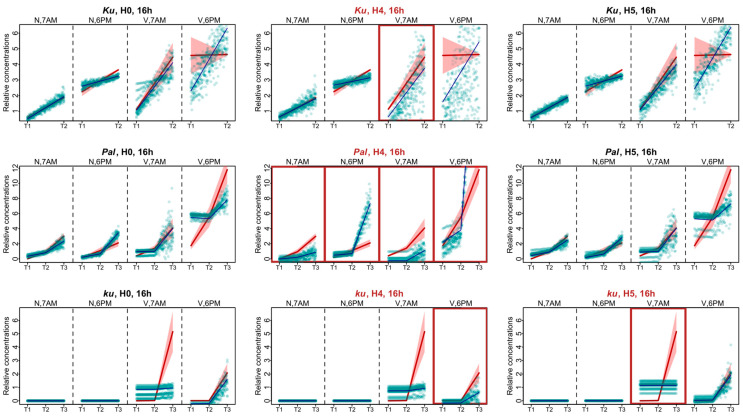
Expression dynamics in Models H4, H5, and H0 compared to experimental data for the 16 h photoperiod. Averaged dynamics of the experimental data are shown in red, and the model solution (average of 1000 runs) is shown in blue. Green dots represent the simulation results from 10 random runs of the minimization process. Models showing specific defects in solutions compared to H0 are marked with brown frames. “N” and “V” stand for non-vernalized and vernalized data; “7 A.M.” and “6 P.M.” are the times of data collection during LD. T1–T4 stand for sampling terms [21].

The experimental data for the 16 h photoperiod had much less points in dynamics than the 8 h data. For these data, in each *L. angustifolius* line, **Model H0** failed to reproduce the *AGL8* expression dynamics for one of the time points after vernalization, being 6 P.M. for the *Ku* and *Pal* lines and 7 A.M. for the *ku* line (Figure 6). As in the SD case, under the 16 h photoperiod, the most severe defects in the fits of **Model H4** compared to **Model H0** were found in the *Pal* line. They manifested themselves both under the non-vernalized conditions and after vernalization (Figure 6). In the *Ku* line, the worst fits of **Model H4** were observed at 7 A.M. at vernalized conditions and were less pronounced than in *Pal* (Figure 6). In the *ku* line under LD, we found the specific defects of *AGL8* patterns in **Model H5** at 7 A.M. and in **Model H4** at 6 P.M. under vernalized conditions. The fitting defects in **Model H4** compared to **Model H0** suggested that the regulatory input of *FTc1* to *AGL8* expression in *ku* increased under LD (Figure 6).

#### 2.2.4. Roles of *FT*-like Genes and Non-*FT* Factors in Models Inferred from the Values of Regulatory Parameters (8 h Photoperiod)

We analyzed parameter values in **Model 1** under the hypotheses H1–H5 and compared them to the hypothesis H0 to uncover the details of regulatory inputs to *AGL8* activation in vernalized and non-vernalized conditions and at different times of day (Supplementary Table S1).

Under SD, the *c*_1_ constant, marking a regulatory contribution of the *FT*-like genes, increased after vernalization in almost all *L. angustifolius lines*. An exclusion of the *FTc1* input in **Model H4** for the *Ku* line resulted in a significant increase in the *c*_1_ value compared to **Model H0**. This was observed in both vernalized and non-vernalized conditions but was most evident after vernalization (Supplementary Table S1). By contrast, in the *Pal* line, **Model H4** showed only a slight increase in the *c*_1_ value after vernalization and a negative *c*_1_ value without vernalization. This suggested that in the *Ku* line under SD, an exclusion of *FTc1* resulted in a significantly increased positive input from other *FT* genes, whereas in *Pal*, this input was minimal or even negative. In the wild *ku* line, the application of **Model H5**, where *AGL8* was regulated only by *FTc1*, led to a slight increase in *c*_1_ in the non-vernalized conditions and very low negative *c1* values after vernalization compared to **Model H0** (Supplementary Table S1). This suggests that the *AGL8* patterning defects in the H5 model are not substantially dependent on the regulation by *FT* genes.

The *c*_0_ constant designates regulation by the non-*FT* factors. In the *Ku* line during the 8 h photoperiod, the differences in *c*_0_ values between **Model H4** and **Model H0** showed a strong dependence on the circadian rhythm. In both vernalized and non-vernalized conditions, *c*_0_ values increased significantly at 3 P.M. but remained unchanged at 9 A.M. compared to the H0 model (Supplementary Table S1). On the contrary, in the *Pal* line, *c*_0_ values in **Model H4** did not significantly depend on vernalization or the circadian rhythm and showed slightly increased values compared with **Model H0** in all conditions and times of the day. In the wild *ku* line, the *c*_0_ constant did not vary significantly between models H5 and H0 with respect to the circadian rhythm. However, the *c*_0_ value in *ku* was higher in the H5 model compared to **Model H0** in vernalized conditions (Supplementary Table S1).

We can conclude that under SD, in the *Ku* line, the defects in fits of **Model H4**, which were dependent on vernalization, compared to H0 (Figure 5), were mostly determined by the *c*_1_ constant. The dependence of fit defects on circadian rhythms was expressed in the variation in *c*_0_ between 9 A.M. and 3 P.M. On the contrary, in the *Pal* line, the substantial fitting defects of **Model H4** (Figure 5) were expressed by a slight increase in the values of *c*_1_ and *c*_0_ constants relative to **Model H0**, which in the case of *c*_1_ depended on vernalization. The vernalization dependence of fitting defects of **Model H5** (Figure 5) in the *ku* line was associated with variation in both *c*_1_ and *c*_0_ constants with respect to vernalization (Supplementary Table S1).

#### 2.2.5. Roles of *FT*-like Genes and Non-*FT* Factors in Models Inferred from the Values of Regulatory Parameters (16 h Photoperiod)

Over the 16 h photoperiod, *c*_1_ values of **Model H4** in the *Ku* line slightly increased after vernalization compared to **Model H0**, while in the *Pal* line, this increase was much more substantial and was observed in the non-vernalized conditions. In the *ku* line after vernalization, *c*_1_ values were slightly increased in Models H2, H4, and H5 after vernalization, with the highest value in **Model H4**. In the non-vernalized data, Models H2 and H5 had negative *c*_1_ values (Supplementary Table S2).

*c*_0_ constant values of **Model H4** in the *Ku* line were elevated compared to **Model H0** across all conditions and times. Interestingly, in the *Pal* line, *c*_0_ values increased in **Model H4** relative to H0 in the non-vernalized conditions at 6 P.M. but decreased in all other conditions and times. In the *ku* line, both Models H2 and H5 showed elevated values of *c*_0_ compared to **Model H0** in all conditions and times of the day, while in **Model H4**, the values of *c*_0_ remained unchanged (Supplementary Table S2).

In conclusion, in the *Ku* line under LD, the defects in **Model H4** compared to H0 (Figure 6) were determined by the increased values of both constants *c*_0_ and *c*_1_, with *c_1_* values dependent on vernalization. The substantial defects of **Model H4** in the *Pal* line were associated with the vernalization dependence of the *c*_1_ constant (Supplementary Table S2). The specific defects in the solution of **Model H4** in the non-vernalized conditions at 6 P.M. were associated with an increase in the *c*_0_ constant (Supplementary Table S2). In the *ku* line, all hypotheses whose cost function values statistically differed from **Model H0** (Figure 4) were associated with the changes in the regulatory constants. *c*_1_ values were slightly elevated in Models H2, H4, and H5 after vernalization, which explained the fitting defects in Models H4 and H5 (Figure 6). The values of *c*_0_ did not depend on vernalization or circadian rhythms (Supplementary Table S2).

### 2.3. Modifications of Model 1

Since **Model 1** had some problems with capturing the *AGL8* expression dynamics, especially for 16 h photoperiod data, we considered several ways to improve the fitting. One constraint of **Model 1** is that the *AGL8* expression level is a linear function of *FT* concentrations, while these concentrations themselves are nonlinear functions of time. We applied the following three modifications of **Model 1**, in which *AGL8* nonlinearly responds to *FT* inputs:

**Model 4:**

$$AGL8=c_{0}+c_{1}{\left(\sum {FT}_{i}\right)}^{2}$$

**Model 5:**

$$AGL8=c_{0}+c_{1}e^{\left(\sum {FT}_{i}\right)}$$

**Model 6:**

$$AGL8=c_{0}+c_{1}log\left(\sum {FT}_{i}\right)$$

We also considered the following modification, in which the model was kept linear but the regulatory coefficient associated with the *FT* influence was assumed to be a linear function of time.

**Model 7:**

$$AGL8=c_{0}+c_{1}t\sum {FT}_{i}.$$

The summation in **Models 4–7** occurs over all *FT*-like genes. It is important that all these modifications have the same number of free parameters as **Model 1**, so that we do not complicate the model structure in terms of parameters and can reliably compare the quality of their fits to data with the baseline **Model 1**.

**Figure 7:**
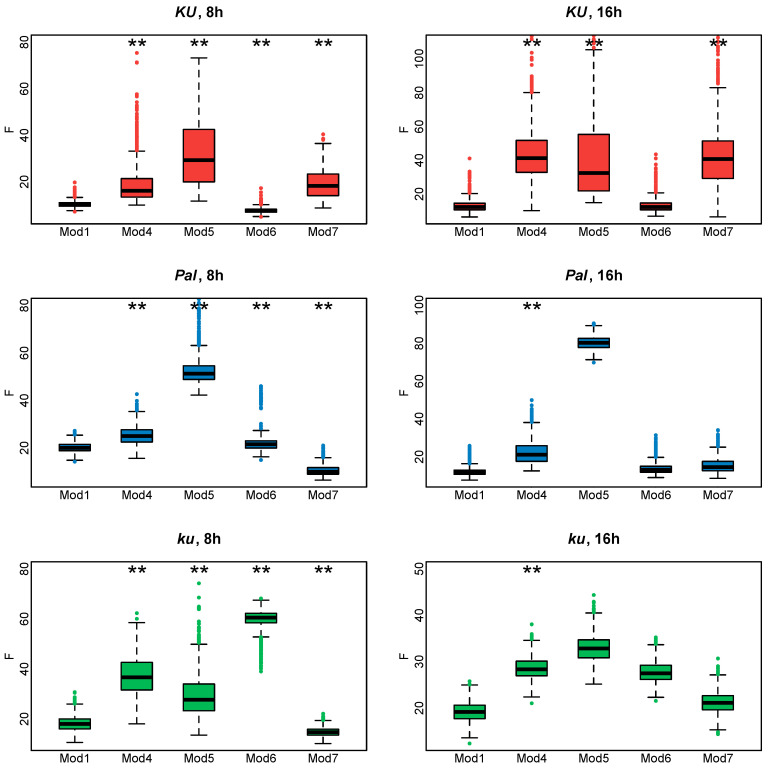
Cost function values (F) for 1000 minimization runs of **Model 1** and **Models 4–7** for three *L. angustifolius* lines. Asterisks indicate statistically significant differences in the mean F between Model i (i = 4…7) and **Model 1** (** *p* < 0.01). The labels 8 h and 16 h are SD and LD photoperiods.

Figure 7 shows the cost function values for the fitting of Models 4–7 to the *AGL8* expression data. It is evident that **Model 6** works better for the *Ku* line, while for the *Pal* and *ku* lines, **Model 7** provides minimal cost function values in both 8 and 16 h photoperiods. However, none of these models outperform **Model 1** equally well in all *L. angustifolius* lines. We concluded that the shortcomings in reproducing *AGL8* expression dynamics by **Model 1** were not due to its lack of nonlinear or time-dependent response to *FT* inputs but presumably to the presence of additional regulatory inputs missing in the model.

## 3. Discussion

In this paper, we analyzed the core regulatory interactions underlying floral transition in the narrow-leafed lupin *L. angustifolius* under variable environmental conditions [21]. The mechanisms of *FT*-like gene activation by vernalization and the photoperiod in *L. angustifolius* are yet unknown, so we used a “hub” approach, assuming that *FT*-like gene expression dynamics reflect the influence of environmental cues.

The narrow-leafed lupin became an interesting object to study mechanisms of floral transition after the discovery of large deletions in the promoter region of the *FTc1* gene. Such deletions caused early flowering and almost abolished the vernalization response [24,26]. Consequently, the *FTc1* gene was considered to confer early flowering and vernalization independence. However, there is yet no direct experimental evidence whether other *L. angustifolius FT*-like genes (*FTa1*, *FTa2*, and *FTc2*) play any role in vernalization-induced flowering.

We suggested a number of hypotheses on how *L. angustifolius FT*-like genes contribute to the regulation of *AGL8*, an ortholog of the *Arabidopsis* core meristem identity genes *AP1* and *FUL* [5,6]. We considered a gene network structure common to many plant species including legumes, in which *FT*-like genes regulate *AP1*-like genes (Figure 1) [8,35,36]. However, in this case, *AGL8* expression was considered in leaves, where it showed strong association with vernalization independence of the *Ku* locus [37]. The expression and function of *AP1* orthologs in leaves have been described in a number of plant species [38,39].

After selecting the most appropriate structure of the regression model, we turned off the regulatory input *L. angustifolius FT*-like genes one by one to figure out their roles in *AGL8* regulation. These numerical experiments could be interpreted as a simulation of loss of function mutations in *FT*-like genes, which have not yet been studied experimentally. Then, we fit the model solutions to *AGL8* expression data in three *L. angustifolius* lines differing in the flowering time and vernalization sensitivity [21]. The greater the deviation of the model solution from the experimental data, the more significant the regulatory role of the missing *FT*-like gene in *AGL8* regulation.

**Figure 8:**
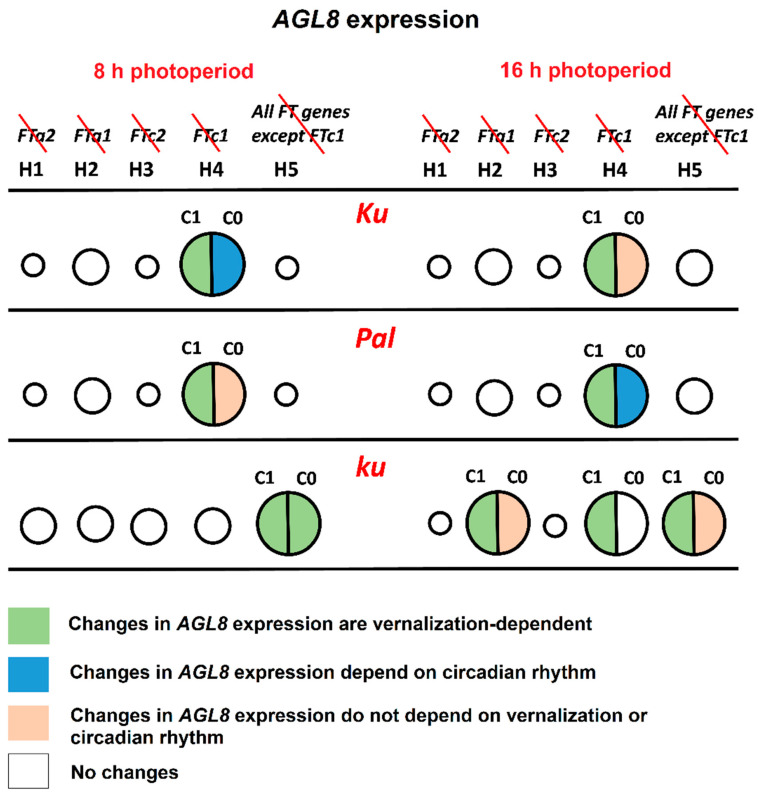
The roles of *FT*-like genes in *AGL8* regulation in *L. angustifolius* models. The figure summarizes the regulatory effects of the exclusion of one or several *FT*-like genes from *AGL8* regulation under 8 and 16 h photoperiods. Circles of different sizes show an effect of *FT*-like gene exclusion on the cost function values of **Model 1** under hypotheses H1–H5. *FT*-like genes excluded from each model are specified in the top panel and crossed out in red. The larger the circle, the stronger the influence of regulators on *AGL8* expression. In models with the smallest circles, cost function values did not show statistically significant differences from model H0, where *AGL8* was regulated by all four *FT*-like genes (*FTa1*, *FTa2*, *FTc1*, and *FTc2*) (Figure 4). Cost function values in the models with middle and large circles had statistically significant differences from model H0. However, only models with large circles exhibited patterning defects and/or changes in regulatory parameters. The association of changes in the regulatory parameters with vernalization and circadian rhythms are indicated by different colors, according to the key at the bottom panel. The *c*_1_ constant presents the regulatory input of *FT*-like genes, while *c*_0_ reflects the regulation of *AGL8* by other factors (Supplementary Tables S1 and S2).

The line carrying the early flowering *Ku* allele showed the most patterning defects under the hypothesis H4, where *FTc1* did not play a role in *AGL8* regulation (Figure 4 and Figure 8). Under SD, an exclusion of *FTc1* from *AGL8* regulation resulted in an increased activation by other *FT*-like genes, especially in vernalized conditions, and by a more intense regulation by other factors, which depended on circadian rhythms (Figure 8, Supplementary Table S1). Under LD, the elimination of *FTc1* regulatory input was associated with a slight increase in regulation by other *FT*-like genes in vernalized conditions and regulation by non-*FT* factors in all conditions and times of the day (Figure 8, Supplementary Table S2). In summary, our models showed that in the *Ku* line, *FTc1* had a maximal effect on *AGL8* expression under both short and long days, although through different mechanisms (Figure 8).

**Figure 9:**
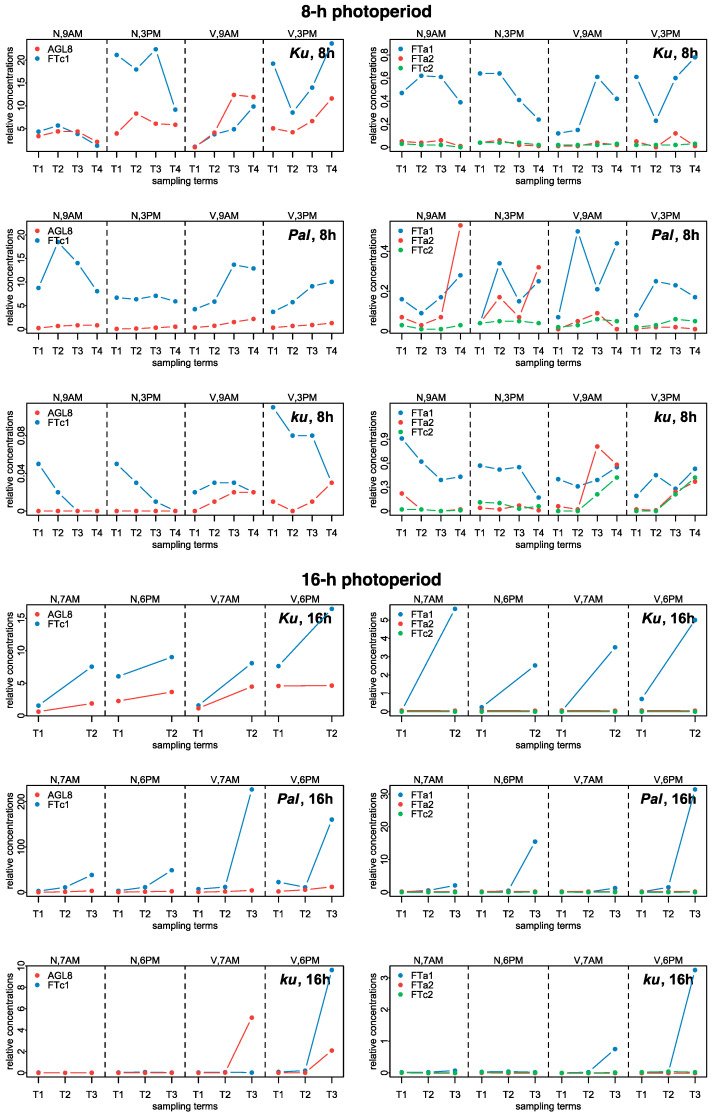
Experimental data on the expression dynamics of *AGL8*, *FTc1*, *FTa1*, *FTa2,* and *FTc2* genes over the 8 h (SD) and 16 h (LD) photoperiods [21]. The data were obtained with qRT-PCR. “N” and “V” stand for non-vernalized and vernalized data; “9 A.M.” and “3 P.M.” are the times of the day when the data were collected during SD, while “7 A.M.” and “6 P.M.” are the times of data collection for LD. T1–T4 stand for sampling terms [21].

In the intermediate *Pal* line, the strongest effect on *AGL8* expression was also provided by the exclusion of the *FTc1* gene under both SD and LD (Figure 4 and Figure 8). However, in this case, the patterning defects were stronger than in *Ku* and depended both on vernalization and circadian rhythms (Figure 5 and Figure 6). The analysis of parameter values suggested that under both SD and LD, an exclusion of *FTc1* resulted in an increased cumulative action of *FTa1*, *FTa2,* and *FTc2* genes, depending on vernalization (Supplementary Tables S1 and S2, Figure 8). Under LD, this exclusion additionally was associated with the action of some other factors, which depended on circadian rhythms (Figure 8). Thus, in both the *Ku* and *Pal* lines, despite differences in their phenology, *AGL8* expression was primarily dependent on *FTc1* without any significant regulatory influence from other *FT*-like genes. For several legume species, the regulatory inputs from vernalization and photoperiod pathways were suggested to be integrated by different *FT*-like genes. For example, in *M. truncatula*, early flowering and vernalization response are provided by the *FTa1* gene, while the *FTb* gene is responsible for photoperiod sensitivity [23,36]. A similar allocation has been reported for *FTb2* and *FTa1* genes in the garden pea *P. sativum* [22]. Given a significant increase in *FTa1* expression in the *L. angustifolius Pal* line under LD (Figure 9), the *FTa1* gene has been proposed to be responsible for photoperiod regulation, in addition to *FTc1*, conferring vernalization response [21]. In our study, an exclusion of *FTa1* from *AGL8* regulation in **Model H2** did not substantially affect cost function or parameter values in the *Pal* line, while it changed the model parameters in the wild *ku* line after vernalization (Figure 4, Supplementary Table S2).

In the wild *ku* line, *AGL8* expression was regulated by different sets of *FT*-like genes compared to *Ku* and *Pal,* and this regulation was dependent on the photoperiod. Under SD, the worst cost function values were observed under the hypothesis H5, where *AGL8* was regulated only by the *FTc1* gene. This resulted in the defects of model fits in the vernalized conditions and in the vernalization dependence of all parameter values (Figure 8, Supplementary Table S1). Thus, under SD, the wild allele of the *FTc1* gene was unable to provide the correct expression of *AGL8* in the absence of regulatory contributions from other *FT*-like genes. Under LD, in addition to the model H5, parameter values were affected in the models H2 and H4 in a vernalization-dependent manner. This demonstrated that under LD, both *FTa1* and *FTc1* genes were necessary for the correct *AGL8* expression in the *ku* allele. Indeed, these regulatory inputs led to floral transition under LD, contrasting with an absence of any strong regulator under SD, where *ku* did not flower [21].

A strong effect of *FTc1* on *AGL8* in the *Ku* and *Pal* lines is explained by its high expression levels, presumably caused by deletions encompassing binding sites of some repressor factors. Despite a large number of candidate transcription factor motifs, some of them were proposed to have functional roles [24,26]. A MADS-box transcription factor AGAMOUS-like 15 (AGL15) has been recently suggested as a candidate regulator of early flowering related to *FTc1* indels in two lupin species, *Lupinus luteus* and *L. angustifolius* [40]. In *Arabidopsis*, AGL15 acts as a floral repressor by binding the *FT* promoter sequence at sites partially overlapping with those bound by the vernalization integrator proteins FLC and SVP [15,40,41]. Interestingly, there is a strong dependence between the number of AGL15 binding sites in the *FTc1* promoter and early flowering of *L. angustifolius*. The wild *ku* allele has five AGL15 repressor binding sites, the intermediate allele *Pal* has two sites, the *Ku* allele has one site, and the *Jul* allele has no AGL15 sites. A similar tendency has been observed for the yellow lupin [40]. The roles of candidate transcription factor binding sites could be further validated with genome editing tools and improved transformation protocols [26,42,43].

In the study [21], the authors examined the expression of two homologs of *A. thaliana* genes involved in the *FLC* vernalization pathway, *CRLK1* and *UGT85A2*. These two genes were selected based on transcriptomic data on their contribution to the vernalization response via *FTc1* in *L. angustifolius* [37]. *CRLK1* functions in the C-repeat binding factor (CBF) cold sensitivity pathway, while *UGT85A2* is involved in the UDP-glycosyltransferase pathway. The downstream genes in these pathways, *CBF* and *CBF EXPRESSION INDUCTOR 1* (*ICE1*), provide regulatory links to *FLC* [44,45]. *CRLK1* showed a negative response to vernalization in early and intermediate *Ku* and *Pal* lines, but a positive or variable response in the wild-type *ku* line. *UGT85A2* was downregulated by vernalization in all *L. angustifolius* lines. The above pathways are putatively underlying the vernalization response related to *FTc1*, a major *FT*-like gene, in *L. angustifolius*.

As in a case of the *FLC* repressor, the *CO* gene, a major integrator of the photoperiod pathway in *Arabidopsis*, does not appear to have a role in *L. angustifolius* [20,21]. Thus, the mechanisms of the photoperiod and circadian rhythm regulation in the narrow-leafed lupin remain challenging. Interestingly, in the *L. angustifolius* lines carrying promoter deletions, the distal promoter regions, which in *Arabidopsis* contain CCAAT binding sites, potentiating CO-mediated activation, remain preserved [21,24,26,46]. However, despite the absence of functional *CO*, many orthologs of *Arabidopsis* photoperiod genes play essential functional roles in temporal legume species, suggesting their possible involvement in *FT* regulation without a central integrator gene [47,48,49,50,51,52].

Our models did not predict any specific function of *FTa2* and *FTc2* genes in *AGL8* regulation. A recent study in the sister lupin species, *L. luteus*, predicted the association of indels in the *FTc2* gene with photoperiod responsiveness with a specific role of a large insertion of a Copia-like retrotransposon element into the *FTc2* third intron [40]. Overall, in this study, the authors considered associations of indels in all four *L. luteus FT*-like genes with phenotypic traits. The application of this approach to *L. angustifolius* could further verify regulatory functions of *FTa1, FTa2,* and *FTc2* genes.

The structure of the genetic network of meristem identity gene regulation by *FT*-like genes (Figure 1) also requires experimental verification. It could be the case that *FTa2* and *FTc2* genes act upstream of *FTc1,* and each gene makes a small contribution into *FTc1* regulation. In this case, *FTc1* acts as a hub, accumulating inputs from all other *FT* genes and transmitting them to *AGL8*. In our models, we considered an overall contribution of each *FT* gene to *AGL8* activation, and we could not judge whether an effect of *FT*-like genes on *AGL8* is direct or indirect. Moreover, it is unclear whether *FT*-like genes form a network of interactions or what the position of each *FT*-like gene is in this network. The application of the reverse genetics methods could provide more details on the gene network structure underlying floral transition in *L. angustifolius*.

To make reliable predictions about the role of each *FT*-like gene, we needed to find the right balance between model complexity and the data structure. Since the experimental data had very poor temporal resolution, we discarded the modeling based on ordinary differential equations and chose the regression-based framework. The linear regression models adequately reflected the dynamics of *AGL8* expression in SD; however, they failed to reproduce *AGL8* data for some conditions under LD. Nevertheless, after testing more complex regression-based models, we concluded that these shortcomings were not due to the nonlinear or time-dependent response of *AGL8* to *FT* inputs but presumably to the presence of additional regulatory inputs that are missing in the model. Despite most models of floral transition considering *FT*-like genes as the sole positive regulators of meristem identity genes [28,29,30,32,33], the role of another integrator gene, *SUPPRESSOR OF OVEREXPRESSION OF CONSTANS (SOC1)*, should also be taken into account. As in a case of the *FT* gene, the *Arabidopsis SOC1* is directly bound by the FLC protein and thus is involved in the vernalization response [53,54]. Legumes have several paralogous copies of *SOC1* genes, which have been recently shown to play a significant role in the vernalization-induced flowering of *M. trancatula* [34,55,56]. The *L. angustifolius SOC1* integrators should be included in the gene network once the experimental data become available.

## 4. Materials and Methods

### 4.1. Experimental Data

The numerical data on gene expression in *L. angustifolius* were obtained from Supplementary Table “Data Sheet 1” of [21] (https://www.frontiersin.org/articles/10.3389/fpls.2020.572135/full#supplementary-material, accessed on 16 December 2024). Three *L. angustifolius* lines were considered, namely *83A:476*, carrying domesticated *FTc1* allele *Ku*, *Palestyna*, carrying *FTc1* allele *Pal* with an intermediate phenology, and *P27255*, carrying the wild *FTc1* allele *ku* [21]. In the paper, for simplicity, we called these lines by the name of the allele. The data presented expression dynamics of *FTa1*, *FTa2*, *FTc1*, *FTc2,* and *AGL8* genes in leaves under 8 h and 16 h photoperiods with and without vernalization. For each of these conditions, plant material was collected at two time points during the day to estimate the dependence of gene expression on circadian rhythms, which were 9 A.M. and 3 P.M. for the 8 h photoperiod and 7 A.M. and 6 P.M. for the 16 h photoperiod [21]. Gene expression dynamics included 2–4 time points (sampling terms) within a period of two weeks prior to flowering (Supplementary Table S5 from [21]). mRNA concentrations (mean ± s.d.) were measured using qRT-PCR with two reference genes (*LanDExH7* and *LanTUB6*). S.d. was a standard deviation of 3 biological replicates, each representing a mean of 3 technical replicates [21]. Figure 9 shows the dynamics of mean mRNA concentration values.

In the early flowering *Ku* line under SD, the levels of expression *FTc1* and *AGL8* were much higher than in *FTa1*, *FTa2,* and *FTc2* and showed slight induction by vernalization prior to flowering. Under LD, the expression of *FTc1* and *AGL8* was nearly independent of vernalization. *FTc1* and *FTa1* concentrations increased prior to flowering in both vernalized and non-vernalized conditions and in *FTa1,* this increase was quite significant. *FTc1* levels were higher in the evening than in the morning under both SD and LD. The expression of *FTa2* and *FTc2* was very low independently of LD or vernalization (Figure 9, [21]).

In the intermediate *Pal* line under SD, the expression of *FTc1* was much higher than in all other genes but was not substantially induced by vernalization contrary to *AGL8*, which showed an increase in vernalized conditions, primarily at 9 A.M. Under LD, the expression of *FTc1* was greatly increased after vernalization independently of the circadian rhythm, while *AGL8* concentration was mostly increased at 6 P.M. The expression of *FTa1* in the *Pal* line under LD showed a very substantial induction in both non-vernalized and vernalized conditions mostly in the evening term. As in the case of *Ku*, the expression of *FTa2* and *FTc2* was very low in the *Pal* line.

In the wild *ku* line under SD, the expression of both *FTc1* and *AGL8* slightly increased after vernalization, while under LD, the expression of *FTc1, FTa1,* and *AGL8* was substantially induced in the vernalized conditions mostly at 6 P.M. Under SD, *FTa2* and *FTc2* genes showed an increased expression after vernalization in contrast to their expression in the *Ku* and *Pal* lines (Figure 9, [21]).

For a more robust parameter estimation process, we performed linear interpolation of the expression values over time and generated additional data points by taking intermediate values from the interpolated function. As a result, 61 data points (means and variances) were obtained in total for the 8 h data and 41 for the 16 h data. Finally, 1000 sets of expression levels were randomly generated by sampling values from the normal distribution with the mean and variance values obtained from the interpolated function.

### 4.2. Models and Parameter Optimization

Regression models of the *AGL8* expression level were applied using expression levels of *FT* genes as predictors, as described in the text. Parameter values in the models were estimated by multiple parameter optimization runs, in which the *AGL8* expression in the model was fitted to that in the data. The fitting was performed by minimizing the following cost function:

$$F=\frac{\sum {\left({AGL8}_{V,9}^{data}-{AGL8}_{V,9}^{model}\right)}^{2}}{\sum {\left(\sigma_{V,9}^{AGL8}\right)}^{2}}+\frac{\sum {\left({AGL8}_{V,3}^{data}-{AGL8}_{V,3}^{model}\right)}^{2}}{\sum {\left(\sigma_{V,3}^{AGL8}\right)}^{2}}\;+\frac{\sum {\left({AGL8}_{N,9}^{data}-{AGL8}_{N,9}^{model}\right)}^{2}}{\sum {\left(\sigma_{N,9}^{AGL8}\right)}^{2}}+\frac{\sum {\left({AGL8}_{N,3}^{data}-{AGL8}_{N,3}^{model}\right)}^{2}}{\sum {\left(\sigma_{N,3}^{AGL8}\right)}^{2}},$$

where “*data*” and “*model*” mark the *AGL8* expression levels in the data and model, respectively; “*V*” and “*N*” mark vernalized and non-vernalized conditions, respectively; and “9” and “3” stand for 9 A.M. an 3 P.M. data, respectively, which indicate times of data collection under SD. Under LD, these times are 7 A.M. and 6 P.M., respectively, but we keep the “9” and “3” notation for simplicity. σ is the standard deviation from the data. The summation in this formula goes over all data points. The numerical minimization procedure was performed in R, using the Nelder–Mead minimization method. The mean and standard deviation of parameter values were estimated from the minimization results obtained for each of the 1000 datasets.

The following Akaike information criterion (AIC) adjusted for small data samples was used to compare the performance of models with different numbers of parameters:

$$AIC=2k-2log\hat{L}+\frac{2k^{2}+2k}{m-k-1},$$

where *k* is the number of parameters, *m* is the number of data points, and $\hat{L}$ is the maximal likelihood. For the weighted cost function *F* used in our study, the maximal likelihood is expressed via the minimal value of *F* as follows: $2log\hat{L}=-F_{min}$ [33].

## Data Availability

All data and modeling results are contained within the article and Supplementary Information.

## Supplementary Materials

The following supporting information can be downloaded at: https://www.mdpi.com/article/10.3390/plants13243548/s1, Figure S1: Dependence of the cost function values on parameter variation for different environmental conditions. Var 1–3 (variants 1–3) represent three different options for variation of parameters in Model 1 under 8 h photoperiod: Var 1: *c*1 differed between vernalized and non-vernalized conditions; Var 2: *c*1 differed between 9 A.M. and 3 P.M.; Var 3: *c*1 did not differ between any conditions. In both *Pal* and *Ku* lines, Var 1 had the lowest value of the cost function. Asterisks indicate statistically significant differences in the mean F between Var 1 and 2 and Var 3 (* *p* < 0.05, ** *p* < 0.01); Table S1: Regulatory parameters for Model 1 under the hypotheses H0-H5 for the 8 h photoperiod. *c*1 constant presents the regulatory input of *FT*-like genes, while *c*0 reflects the regulation of *AGL8* by other factors. The values of each constant differ between vernalized (V) and non-vernalized (N) conditions. *c*0 values also vary depending on the time of day: “9” corresponds to the data collected at 9 A.M. and “3” to the data collected at 3 P.M. [21]; Table S2: Regulatory parameters for Model 1 under the hypotheses H0-H5 for the 16 h photoperiod. *c*1 constant presents the regulatory input of *FT*-like genes, while *c*0 reflects the regulation of *AGL8* by other factors. The values of each constant differ between vernalized (V) and non-vernalized (N) conditions. *c*0 values also vary depending on the time of day: “7” corresponds to the data collected at 7 A.M. and “6” to the data collected at 6 P.M. [21].
