# Supplementary material for: Modeling Floral Induction in the Narrow-Leafed Lupin Lupinus angustifolius Under Different Environmental Conditions
Source: Plants (Basel). 2024 Dec 19;13(24):3548. doi: 10.3390/plants13243548 (PMC11678331; doi:10.3390/plants13243548)
Supplement: Supplementary file 1 [file plants-13-03548-s001.zip › plants-3309053-supplementary.pdf]

## 1. Supplementary Figures

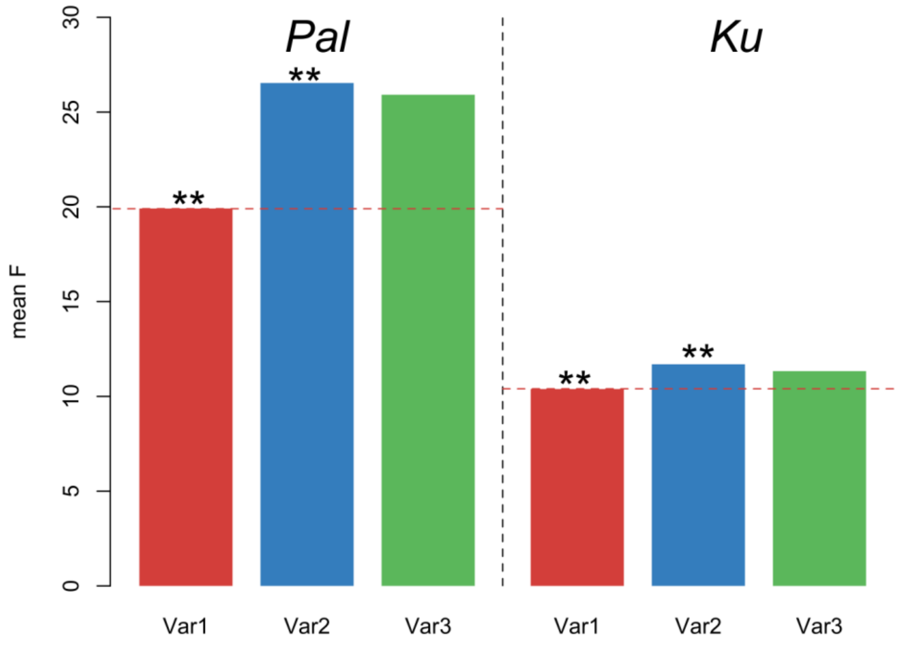


**Supplementary Figure S1.** Dependence of the cost function values on parameter variation for different environmental conditions. Var 1-3 (variants 1-3) represent three different options for variation of parameters in Model 1 under 8 h photoperiod: Var 1: *c1* differed between vernalized and non-vernalized conditions; Var 2: $c_1$ differed between 9 A.M. and 3 P.M.; Var 3: $c_1$ did not differ between any conditions. In both *Pal* and *Ku* lines, Var 1 had the lowest value of the cost function. Asterisks indicate statistically significant differences in the mean F between Var 1 and 2 and Var 3 (* p< 0.05, ** p<0.01).

## 2. Supplementary Tables

| Regulatory constant | Line | H0 | H1 | H2 | H3 | H4 | H5 |
|---|---|---|---|---|---|---|---|
| | *Ku* | 2.397 | 2.382 | 2.602 | 2.382 | 2.002 | 2.607 |
| ***c0*,N9** | *Pal* | 0.4492 | 0.497 | 0.3773 | 0.4746 | 0.8558 | 0.4289 |
| | *ku* | 0.001169 | 0.0008705 | 0.000953 | 0.0009365 | 0.001046 | 0.0009344 |
| | *Ku* | 0.3659 | 0.2756 | 0.6939 | 0.2802 | 3.403 | 0.6562 |
| ***c0*,N3** | *Pal* | 0.1477 | 0.1736 | 0.1066 | 0.1593 | 0.4411 | 0.1331 |
| | *ku* | 0.001177 | 0.0008736 | 0.0009204 | 0.0009559 | 0.001049 | 0.0008986 |
| | *Ku* | 3.391 | 3.42 | 4.05 | 3.419 | 3.522 | 3.99 |
| ***c0*,V9** | *Pal* | 0.04384 | 0.05069 | 0.04252 | 0.03259 | 0.81 | 0.02889 |
| | *ku* | 0.0007918 | 0.005291 | 0.002591 | -0.002503 | 0.001421 | 0.01507 |
| | *Ku* | -4.749 | -4.668 | -3.94 | -4.726 | 1.475 | -4.057 |
| ***c0*,V3** | *Pal* | -0.109 | -0.1086 | -0.08387 | -0.1128 | 0.2854 | -0.08985 |
| | *ku* | -0.004392 | -0.005443 | 0.0006515 | -0.005418 | -0.002895 | 0.01531 |
| | *Ku* | 0.3026 | 0.3076 | 0.2947 | 0.307 | 2.902 | 0.2978 |
| ***c1*,N** | *Pal* | 0.01259 | 0.009162 | 0.01961 | 0.01103 | -0.2959 | 0.01603 |
| | *ku* | -0.0002681 | 0.0001591 | 0.0005285 | 5.137e-05 | -7.302e-05 | 0.003742 |
| | *Ku* | 0.7229 | 0.7213 | 0.6933 | 0.7226 | 6.962 | 0.704 |
| ***c1*,V** | *Pal* | 0.1145 | 0.1146 | 0.1191 | 0.1156 | 0.8735 | 0.1209 |
| | *ku* | 0.01662 | 0.0215 | 0.02604 | 0.0212 | 0.01635 | -0.06648 |

**Supplementary Table S1**. Regulatory parameters for Model 1 under the hypotheses H0-H5 for the 8 h photoperiod. $c_1$ constant presents the regulatory input of *FT*-like genes, while $c_0$ reflects the regulation of *AGL8* by other factors. The values of each constant differ between vernalized (V) and non-vernalized (N) conditions. $c_0$ values also vary depending on the time of day: "9" corresponds to the data collected at 9 A.M. and "3" to the data collected at 3 P.M. [21].

| Regulatory constant | Line | H0 | H1 | H2 | H3 | H4 | H5 |
|---|---|---|---|---|---|---|---|
| | *Ku* | 0.3246 | 0.3154 | 0.258 | 0.3278 | 0.6296 | 0.258 |
| ***c0*,N7** | *Pal* | 0.2073 | 0.2043 | 0.2861 | 0.2063 | -0.0416 | 0.2831 |
| | *ku* | -0.0009 | -0.0008 | 0.004 | -0.0009 | -0.0009 | 0.0035 |
| | *Ku* | 1.8329 | 1.8169 | 1.3076 | 1.832 | 2.6148 | 1.3066 |
| ***c0*,N6** | *Pal* | 0.0478 | 0.0401 | 0.015 | 0.0447 | 0.4846 | 0.0049 |
| | *ku* | 0.0011 | 0.0011 | 0.0085 | 0.0011 | 0.0011 | 0.0061 |
| | *Ku* | 0.5684 | 0.545 | 0.3751 | 0.5635 | 0.6202 | 0.3862 |
| ***c0*,V7** | *Pal* | 0.8443 | 0.8405 | 0.8129 | 0.8407 | -0.3328 | 0.8022 |
| | *ku* | 0.8489 | 0.8393 | 1.1559 | 0.8462 | 0.7423 | 1.1439 |
| | *Ku* | -0.2448 | -0.2844 | -0.9892 | -0.2556 | 0.9747 | -0.9681 |
| ***c0*,V6** | *Pal* | 5.1471 | 5.136 | 5.0013 | 5.1455 | 2.1051 | 4.9705 |
| | *ku* | -0.238 | -0.2334 | 0.0194 | -0.2429 | -0.1737 | 0.0207 |
| | *Ku* | 0.1227 | 0.1242 | 0.2156 | 0.1225 | 0.21 | 0.2159 |
| ***c1*,N** | *Pal* | 0.0532 | 0.0534 | 0.0575 | 0.0533 | 0.4418 | 0.0577 |
| | *ku* | 0.0014 | 0.0014 | -0.0804 | 0.0014 | 0.0014 | -0.0646 |
| | *Ku* | 0.3061 | 0.3087 | 0.4472 | 0.3069 | 0.8917 | 0.4457 |
| ***c1*,V** | *Pal* | 0.0138 | 0.0138 | 0.0143 | 0.0138 | 1.1217 | 0.0143 |
| | *ku* | 0.1432 | 0.1429 | 0.1956 | 0.1441 | 0.2433 | 0.1958 |

**Supplementary Table S2**. Regulatory parameters for Model 1 under the hypotheses H0-H5 for the 16 h photoperiod. $c_1$ constant presents the regulatory input of *FT*-like genes, while $c_0$ reflects the regulation of *AGL8* by other factors. The values of each constant differ between vernalized (V) and non-vernalized (N) conditions. $c_0$ values also vary depending on the time of day: "7" corresponds to the data collected at 7 A.M. and "6" to the data collected at 6 P.M. [21].
